# Radiation therapy combined with intracerebral administration of carboplatin for the treatment of brain tumors

**DOI:** 10.1186/1748-717X-9-25

**Published:** 2014-01-14

**Authors:** Weilian Yang, Rolf F Barth, Tianyao Huo, Robin J Nakkula, Michael Weldon, Nilendu Gupta, Lawrence Agius, John C Grecula

**Affiliations:** 1 Department of Pathology, The Ohio State University, Columbus, OH 43210, USA; 2 Department of Radiation Oncology, The Ohio State University, Columbus, OH 43210, USA; 3 Department of Pathology, Mater Dei Hospital, University of Malta Medical School, Msida, Malta; 4 Current address: Department of Health Outcomes and Policy, College of Medicine, University of Florida, Gainesville, FL, USA

**Keywords:** F98 glioma, Carboplatin, Convection enhanced delivery, Radiotherapy, Brain tumors

## Abstract

**Background:**

In this study we determined if treatment combining radiation therapy (RT) with intracerebral (i.c.) administration of carboplatin to F98 glioma bearing rats could improve survival over that previously reported by us with a 15 Gy dose (5 Gy × 3) of 6 MV photons.

**Methods:**

*First,* in order to reduce tumor interstitial pressure, a biodistribution study was carried out to determine if pretreatment with dexamethasone alone or in combination with mannitol and furosemide (DMF) would increase carboplatin uptake following convection enhanced delivery (CED). *Next,* therapy studies were carried out in rats that had received carboplatin either by CED over 30 min (20 μg) or by Alzet pumps over 7 d (84 μg), followed by RT using a LINAC to deliver either 20 Gy (5 Gy × 4) or 15 Gy (7.5 Gy × 2) dose at 6 or 24 hrs after drug administration. *Finally,* a study was carried out to determine if efficacy could be improved by decreasing the time interval between drug administration and RT.

**Results:**

Tumor carboplatin values for D and DMF-treated rats were 9.4 ±4.4 and 12.4 ±3.2 μg/g, respectively, which were not significantly different (P = 0.14). The best survival data were obtained by combining pump delivery with 5 Gy × 4 of X-irradiation with a mean survival time (MST) of 107.7 d and a 43% cure rate *vs.* 83.6 d with CED *vs.* 30-35 d for RT alone and 24.6 d for untreated controls. Treatment-related mortality was observed when RT was initiated 6 h after CED of carboplatin and RT was started 7 d after tumor implantation. Dividing carboplatin into two 10 μg doses and RT into two 7.5 Gy fractions, administered 24 hrs later, yielded survival data (MST 82.1 d with a 25% cure rate) equivalent to that previously reported with 5 Gy × 3 and 20 μg of carboplatin.

**Conclusions:**

Although the best survival data were obtained by pump delivery, CED was highly effective in combination with 20 Gy, or as previously reported, 15 Gy, and the latter would be preferable since it would produce less late tissue effects.

## Background

Although cisplatin and carboplatin are highly effective anti-cancer drugs, which have been used clinically to treat a variety of malignancies, they have not been effective in patients with high grade gliomas [1]. Despite the fact that their tumoricidal activity can be demonstrated *in vitro*[2], their systemic toxicity, high water solubility, and inability to effectively penetrate the blood-brain barrier (BBB) have limited their usefulness for treating patients with brain tumors. A European Organization for Research and Treatment of Cancer (EORTC) clinical trial in patients with supratentorial malignant gliomas, who had received a combination of cisplatin and RT, failed to demonstrate any improvement in either progression free or overall survival [3]. This brought to an end any further clinical studies to investigate the combination of platinum (Pt) compounds and photon radiation to treat high grade gliomas.

Extensive experimental studies have been carried out by Elleaume and her research team in France [4-8] and by us in the United States [9] to evaluate intracerebral (i.c.) delivery of either cisplatin or carboplatin in combination with RT. This combination has produced the best survival data that ever have been reported with the F98 rat glioma model [10]. The purpose of the present study was to determine if therapeutic efficacy could be improved over that previously reported by [9] using two different RT regimens in combination with i.c. administration of carboplatin by either convection enhanced delivery (CED) or Alzet pump delivery. Both of these approaches can deliver a therapeutic agent directly to the site of the brain tumor, completely bypassing the BBB and allowing one to obtain a drug concentration that can be up to 1,000× greater than that achieved by systemic administration of carboplatin [9]. In the present study the best survival data and cure rates were obtained by administering carboplatin via Alzet osmotic pumps or CED in combination with a 5 Gy × 4 fractionated dose of 6 MV photons with no treatment-related mortality. However, equivalent survival data were reported previously by us [9] using a fractionated 15 Gy dose (5 Gy × 3), and the latter appears to be the optimum regimen since it would reduce long term radiation related effects on the brain.

## Methods

### F98 rat glioma model

The F98 rat glioma has been propagated *in vitro* and *in vivo* since 1971 and, as described in a recent review [10], it has been used in a wide variety of studies in experimental neuro-oncology. It is a radioresistant tumor that is invariably fatal with an inoculum of as few as 100 cells. F98 cells were grown in Dulbecco’s modified Eagle’s medium (DMEM) containing 5% fetal bovine serum, as previously described [9]. All animal studies were carried out in accordance with the *Guide for the Care and Use of Laboratory Animals* (National Academy Press, Washington, DC, 1996) and the protocol was approved by the Institutional Laboratory Animal Care and Use Committee. Male Fischer rats (Animal Production Branch, National Cancer Institute, Frederick, MD) weighing ~200–220g were used in the present study. A stereotactic implantation procedure was employed [11]. F98 glioma cells were suspended in DMEM containing low gelling temperature agarose at a concentration of either 10^3^ cells/10 μl for therapy studies or 10^5^ cells/10 μl for biodistribution studies. These were injected into the right caudate nucleus over 10-15 sec.

### Biodistribution of carboplatin in F98 glioma bearing rats

A greater number of cells were used for biodistribution studies in order to have a larger tumor mass for determination of carboplatin concentrations. In order to reduce tumor interstitial pressure [12], a biodistribution study was carried out to determine if pretreatment with dexamethasone (D), alone or in combination with mannitol and furosemide (DMF) would increase the tumor uptake of carboplatin in F98 glioma bearing rats following i.c. convection enhanced delivery (CED) compared to untreated rats. Eleven to 13 d after tumor implantation, biodistribution studies were initiated in either untreated rats or those that had received either intraperitoneal (i.p.) D (3 mg/kg b.w.) daily × 3 alone or in combination with intravenous (i.v.) M (2.5 g/kg b.w.) at 0.25 ml/min over 10 min and i.v. F (2 mg/kg b.w.) over 15 min. As reported by Boucher et al., this regimen produced a marked reduction in the intratumoral interstitial fluid pressure in F98 glioma bearing rats [12]. Carboplatin (Hospira Inc. Lake Forrest, IL) was administered by CED (0.33 μl/min over 30 min) [9]. Immediately following termination of CED, samples of blood were taken and the animals were euthanized, their brains were removed, tumors were carefully dissected out, weighed, frozen, and stored at −20°C and eventually processed for Pt determinations by means of inductively coupled plasma–optical emission spectroscopy (ICP-OES) [9]. Based on the atomic weight of Pt (195.1), the concentrations of carboplatin (M.W. 371 Da) were calculated by multiplying the Pt values by 1.90.

### Therapy studies

The first set of experiments was carried out using a 20 μg dose of carboplatin, administered i.c. by CED (0.33 μl/min) over 30 min at either 7 d or 13 d after tumor cell implantation or alternatively by infusion using Alzet osmotic pumps (model #2001, Durect Corp., Cupertino, CA) over 7 d (84 μg in 168 μl at 1 μl/hr). In order to determine if the time interval between drug administration and RT would improve survival times, rats were irradiated at either 6 or 24 hr later with 6 MV photons using a Siemens Mevatron linear accelerator. The rats were irradiated under continuous isoflurane anesthesia in a specially fabricated circular plexiglas chamber divided into 4 compartments radiating out from the center. The animals’ bodies were shielded using a pinwheel-shaped cerrobend block so that only the right cerebral hemisphere of their heads, which were pointed inwards towards the center, was irradiated. The chamber had an inlet and outlet for administration of isoflurane and oxygen via an anesthesia machine (model Aestiva/5 7900, GE Datex-Ohmeda). The tumor bearing right cerebral hemisphere was irradiated in either four 5 Gy or two 7.5 Gy fractions. The radiation dose was calculated to mid-point of the brain along the anterior-posterior axis. In the second study, rats received two divided doses of carboplatin (10 μg in 20 μl) by CED on days 13 and 15 following tumor implantation and they were irradiated with 7.5 Gy on days 14 and 16.

### Evaluation of therapeutic response

All experimental animals were weighed 3 times per week and their clinical status was evaluated at the same time. Once the animals had progressively growing tumors, as evidenced by sustained weight loss (20% of their body weight following treatment) they were euthanized by exposure to CO_2_. Survival times were determined by adding 1-2 days to the time between tumor implantation and euthanization. Rats surviving > 180 d were designated as “cured” and were euthanized. The brains of all animals in the therapy studies were removed after death, and processed for neuropathologic examination, stained with hematoxylin and eosin (H&E), and examined microscopically.

### Neuropathologic evaluation of glioma bearing rats following CED of carboplatin and X-irradiation

Four groups, consisting of 6–7 tumor bearing rats each, received CED of carboplatin (20 μg in 10 μl) on d 13 or 14 following implantation of 10^4^ F98 glioma cells and were irradiated with four 5 Gy fractions beginning 24 hr following CED. Cohorts of 6–7 rats were euthanized at 1, 2, 3, or 4 weeks following termination of X-irradiation (25, 32, 39, and 46 d following implantation). Their brains were removed, fixed in 10% buffered formalin, sectioned coronally, and then processed for neuropathologic examination.

### Statistical evaluation of data

The mean carboplatin concentrations ± standard deviations were computed for tumor and the right and left cerebral hemispheres for both untreated and DMF-treated animals. A two-tailed, two sample t test was used to determine statistical significance (P-value ≤0.05). To study the effects of treatment on survival, the MST, standard error (SE), and median survival time (MeST) were calculated for each group and Kaplan-Meier survival curves were plotted. An overall Log Rank test was performed to test for equality of survival curves over the six groups, and between individual groups [13]. The overall α level was 0.05, and multiple comparisons were adjusted using the Bonferroni correction [14]. The percent increased life span was determined, as previously described [15]. In order to determine the sample size that would have been required to demonstrate statistical significance following CED of carboplatin to untreated and DMF-treated rats, sample size calculations were carried out using SAS 9.3 (SAS Institute, Cary, NC). Proc Power procedure with a one-way ANOVA. Sample sizes were calculated with power = 0.8, based on the group means and pooled standard deviation of the Pt concentration in tumor of untreated rats vs DMF-treated rats with an α level = 0.05.

## Results

### Biodistribution studies

The biodistribution data for two groups of eight F98 glioma bearing rats that had received 20 μg of carboplatin either alone or following pre-treatment with either D alone or DMF are summarized in Table 1. Since the carboplatin values following the administration of dexamethasone D alone or DMF were equivalent, these values were combined. The mean tumor values and ranges for rats that received carboplatin alone were 9.41 ±4.40 μg/g and 3.02–17.10 μg/g, respectively. The corresponding values for rats pre-treated with DMF were 12.43 ±3.17 μg/g and 8.55–18.51 μg/g. Although these differences in carboplatin values were not significant (P = 0.14) there was a suggestion that DMF treatment increased the tumor carboplatin concentrations. In contrast to the broad ranges that were seen in the tumor carboplatin values, the normal brain values for the right (tumor bearing) and left cerebral hemispheres were all in a very narrow range. The carboplatin values in normal brain of untreated *vs.* DMF treated rats were not significantly different (P = 0.22 and 0.52 for right and left cerebral hemispheres, respectively).

**Table 1:** **Biodistribution of carboplatin in F98 glioma bearing rats following CED in either untreated or dexamethasone, mannitol, and furosemide treated rats**^**a**^

	**Carboplatin concentration (μg/g tissue)**^**b**^					
**Animal no.**	**Untreated**			**DMF treated**		
	**Tumor**	**R Brain**	**L Brain**	**Tumor**	**R Brain**	**L Brain**
1	3.02	1.41	1.35	8.55	1.63	1.01
2	6.34	0.82	0.45	10.16	1.22	0.78
3	7.92	1.55	1.00	10.63	1.83	1.12
4	8.15	1.13	0.92	10.64	1.79	0.83
5	8.84	1.62	0.88	12.85	3.10	1.34
6	9.84	0.92	0.30	13.32	0.95	0.99
7	14.10	2.05	0.84	14.81	1.30	0.82
8	17.10	1.74	1.34	18.51	2.28	0.96
Mean ± SD^c^	9.41 ±4.40	1.41 ±0.42	0.89 ±0.37	12.43 ±3.17	1.76 ±0.68	0.98 ±0.18
Median	8.50	1.48	0.90	11.75	1.71	0.98
Tumor: brain ratios		6.7:1	10.6:1		7.1:1	12.7:1

^a^Rats received either i.p. dexamethasone (D) (3 mg/kg b.v. daily for 3 days) or i.p. dexamethasone (D) followed by i.v. mannitol (M) (2.5 g/kg b.w.) at 0.25 ml/min over 10 min, followed 15 min later by i.v. furosemide (F) (2 mg/kg b.w.). Since the carboplatin values were equivalent, these were combined.

^b^Rats were euthanized at ~20 min following termination of CED, their brains removed, tumors dissected out, as well as the remainder of the tumor bearing right and non-tumor bearing left cerebral hemispheres. Platinum concentrations were determined by means of ICP-OES and then converted to carboplatin by multiplying by 1.9.

^c^SD designates the standard deviation.

### Therapeutic efficacy of X-irradiation in combination with i.c. carboplatin

**Figure 1:**
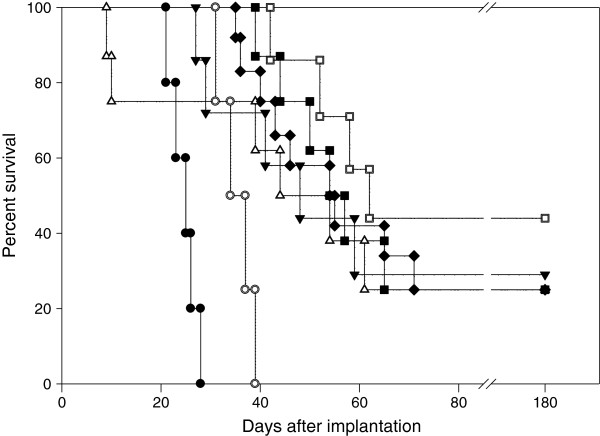
**Kaplan-Meier survival plots of F98 glioma bearing rats following administration of carboplatin by CED or Alzet pumps in combination with X-irradiation.** Survival times in days after implantation have been plotted for untreated animals (●), X-irradiation only (5 Gy × 4) (○), CED of carboplatin (20 μg) + X-irradiation 6 h later (▼), CED of carboplatin (20 μg) at 7 d + X-irradiation (Δ), CED of carboplatin (20 μg) + X-irradiation 24 h later (■), carboplatin (84 μg) administered by Alzet pumps + X-irradiation (□), and CED of carboplatin (10 μg × 2) on days 13 and 15 + X-irradiation (7.5 Gy × 2) on days 14 and 16 (♦).

In the first series of experiments, a radiation dose of 20 Gy in four 5 Gy fractions was administered to the right, tumor bearing cerebral hemisphere in combination with 20 μg of carboplatin, administered by CED or 84 μg by Alzet pump infusion. The survival data are summarized in Table 2 and Kaplan-Meier survival plots are shown in Figure 1. The best survival data with no treatment-related toxicity were seen in rats that received carboplatin via Alzet pumps between days 7 and 13 following tumor implantation with a MST of 107.7 ±20.7 d and a cure rate (i.e., survival > 180 d) of 43%. Rats that received carboplatin by CED on day 13, followed 24 hrs later by the initiation of RT, had a MST of 83.6 ± 21.5 d, with a 25% cure rate. If RT was initiated 6 hrs following CED, the MST was 80.6 ± 18.5 d. However, there were 2 of 8 treatment-related early deaths (d 27and 29) and if these were excluded, the cure rate was 29%. If administration of carboplatin by CED was carried out on d 7 following implantation and RT was initiated on d 8, the MST was 72.1 ± 17.3 d and a cure rate of 25% with 2 or 8 early treatment-related deaths (d 9 and 10), which were attributed to cerebral edema. In contrast, untreated controls had a MST of 24.6 ± 1.1 d and X-irradiated rats had an MST of 35.3 ± 1.8d, both within a very narrow range compared to rats that received RT in combination with carboplatin, which had very broad ranges. Using the Log Rank test, the overall difference between the various treatment groups and untreated controls was highly significant (P < 0.0001), as were the differences between animals that were X-irradiated vs. those that received combination therapy (P ≤0.003).

**Table 2:** Survival data of F98 rats following CED of carboplatin in combination with X-irradiation

		**Survival times (days)**^**b**^			**% Increased life span**	
**Treatment group**^**a**^	**N**	**Mean ± SE**	**Median**	**Range**	**Mean**	**Median**
Untreated controls	5	24.6 ±1.1	25	21–28	0	0
X-irradiation	5	35.3 ±1.8	35.5	31–39	43	42
CED of carboplatin (on day 13) + X-irradiation (6 h later)	8^c^	80.6 ±18.5^c^	48	27- > 180(2)	227	92
CED of carboplatin (on day 13) + X-irradiation	8	83.6 ±21.5	55.5	39- > 180(2)	240	122
CED of carboplatin (on day 7) + X-irradiation	8^d^	72.1 ±17.3^d^	49	9- > 180(2)	193	96
Alzet pump delivery^e^ (d 7-13) + X-irradiation	8	107.7 ±21	62	42- > 180(3)	337	148

^a^Stereotactic intracerebral implantation of 10^3^ F98 glioma cells was carried out on d 0. Carboplatin (20 μg in 10 μL) was administered by convectionenhanced delivery at a flow rate of 0.33 μL/min. for 30 min. on d 13 following implantation. Alternatively, they received 84 μg of the drug by Alzet pumps over 7 d (168 μl at 1 μl/h). Animals received 20 Gy of 6 MV LINAC X-rays, delivered in 5 Gy fractions beginning 24 hrs after CED unless otherwise indicated.

^b^N indicates the number of animals per group and SE designates the standard error. Survival of >180 d indicates that these animals have been cured of their tumors. The number in parenthesis indicates the number of long-term survivors.

^c^Three treatment-related early deaths on d 27 and 29 were included in these calculations.

^d^Two treatment-related early deaths on d 9 and 10 were included in these calculations.

^e^Carboplatin was administered by Alzet pump (84 μg over 168 hr) followed 24 hr later by X-irradiation.

In the second series of experiments, a 15 Gy dose (7.5 Gy × 2 fraction) in combination with carboplatin (10 μg/d × 2 d) was carried out to determine if the survival data could be improved if the carboplatin dose was divided so that a higher drug concentration would be available at the time that RT was initiated. These data are summarized in Table 3 and Figure 1. The MST of these rats was 82.1 ±15.5 d with a 25% cure rate and no treatment-related early deaths. Rats that received a divided dose of carboplatin alone by CED had a MST of 46.8 ±7.5 d, and those that only had received RT had a MST of 30.0 ±1.9 d (P = 0.0006). Neuropathologic examination of the brains of the non-surviving rats that received carboplatin by either CED or Alzet pump delivery, followed by X-irradiation, revealed both macroscopic and microscopic deposits of tumor at the times of their deaths. In contrast, the brains of the long-term survivors (>180 d) showed no microscopic evidence of tumor.

**Table 3:** Survival time of rats bearing F98 gliomas following CED of carboplatin (10 μg × 2) and 15 Gy X-irradiation (7.5 Gy × 2)

		**Survival times (days)**^**c**^			**% Increased life span**	
**Treatment group**^**a**^	**N**	**Mean ± SE**	**Median**	**Range**	**Mean**	**Median**
Untreated	5	24.6 ± 1.1	25	21–28	0	0
X-irradiation^b^	4	30.0 ± 1.9	30	28–32	22	20
CED of carboplatin	5	46.8 ± 7.5	43	33–67	90	72
CED of carboplatin + X-ray	12	82.1 ± 15.5	54.5	35 > 180(3)	234	118

^a^Stereotactic intracerebral implantation of 10^3^ F98 glioma cells was carried out on d. 0. Carboplatin (10 μg in 10 μL) was administered by convection enhanced delivery at a flow rate of 0.33 μL/min. for 30 min on day 13^th^ and 15^th^.

^b^Animals received 15 Gy of 6 MV LINAC X-rays, delivered in two 7.5 Gy fractions on day 14^th^ and 16^th^.

^c^Survival of >180 d. indicates that these animals have been cured of their tumors. The number in parenthesis indicates the number of long-term survivors.

A summary of the radiation dosing schedules and the equivalent doses compared to conventional 2 Gy fractions are shown in Table 4. Based on these calculations, a 15 Gy dose in three 5 Gy fractions, as previously reported [9], was the best in terms of normal tissue sparing and produced identical MSTs in F98 glioma bearing rats compared to 20 Gy in four 5 Gy fractions (83.4 d vs. 83.6 d, respectively).

**Table 4:** Comparison of radiation dosing paradigns

**Equivalent dose**^**a**^	**Physical dose and fractionation regimen**^**a**^		
Total dose	15 Gy (5 Gy × 3)^b^	15 Gy (7.5 Gy × 2)	20 Gy (5 Gy × 4)
Acute responding tissues (α/β = 10)	18.75 Gy	21.88 Gy	25 Gy
Late responding tissues (α/β = 2)	26.25 Gy	35.63 Gy	35 Gy

^a^Using an α/β ratio of 10 for acute (tumor) effects and 2 for late (normal brain) effects, the doses indicated were at conventional 2 Gy fractions.

^b^Dose regimen utilized in our previously published data [9].

### Neuropathologic evaluation following combination therapy

**Figure 2:**
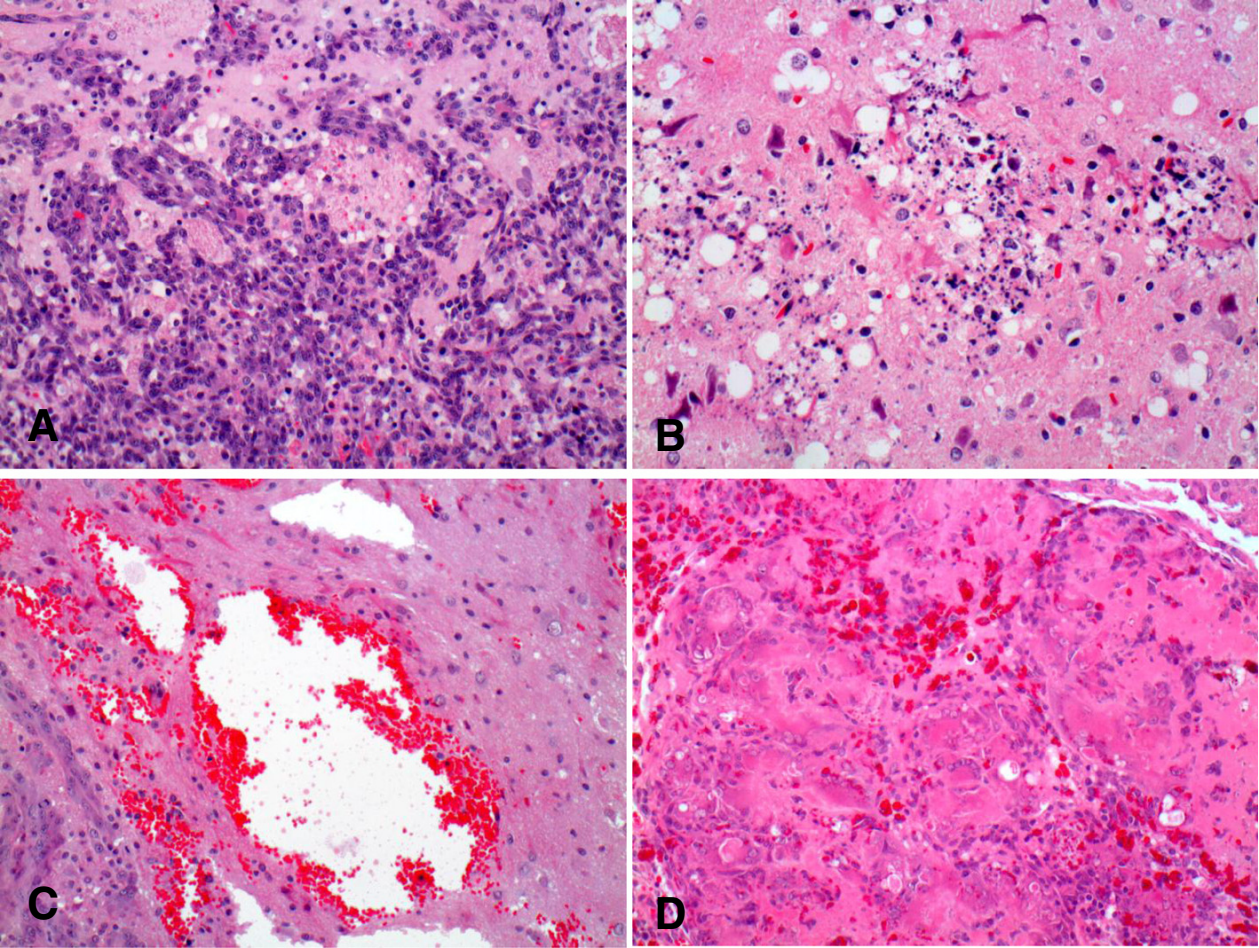
**Neuropathologic correlates. (A)** Brain of an untreated F98 glioma bearing rat that died on d. 26 following tumor implantation. The tumor measured 4 mm in diameter, had a central zone of necrosis, and was highly infiltrative of normal brain. **(B)** Brain of an F98 glioma bearing rat that received 20 μg of carboplatin by CED, followed 24 hrs later by the first of four 5 Gy fractions of 6 MV photons, and was euthanized one week later (25 d after tumor implantation). No tumor cells were identified in this and two additional sections. There is a focal area of gliosis. The superficial white matter shows debris, which possibly could have represented tumor cells that had been killed as a result of treatment, scattered lymphocytes and macrophages, and prominent white matter vacuolation. Otherwise, the remainder of the brain was histologically unremarkable. **(C)** Brain of an F98 glioma bearing rat that received four 5 Gy fractions, administered on days 14, 15, 16 and 17 following tumor implantation and died on d 34. The tumor measured 5 mm with a prominent, necrotic central core and was infiltrative of surrounding white matter. There are scattered dilated (ectatic) vessels and prominent areas of hemorrhage, both of which may be radiation related. **(D)** Brain of an F98 glioma bearing rat that received 20 μg of carboplatin on d 7 after implantation followed by four 5 Gy fractions of 6 MV photons and died on d. 44. The tumor measured 3 mm with a central necrotic core. There are focal collagen deposits with hyalinization and multiple microscopic foci of hemorrhage, which most likely are radiation related changes. All sections were stained with H & E and photographed at a magnification of 200x.

At one week following administration of carboplatin (20 μg) by CED and termination of X-irradiation (5 Gy × 4), the brains of only 2 of 7 rats were microscopically positive for tumor. However, it should be pointed out that since as few as 100 tumor cells can be fatal, it is more than likely than not that a high percentage of these animals would have died of their brain tumors if they had been followed for a longer period of time. The brains of the other rats showed focal changes consisting of gliosis, scattered infiltrates of lymphocytes and macrophages (Figure 2B) compared to those of untreated rats (Figure 2A), which had highly infiltrative tumors. At 2 weeks, the brains of 5 of 6 rats were microscopically positive for tumor. However, there was considerable variability in the anatomic locations of these microscopic foci of tumor cells, including subcortical, leptomeningeal, and the wall of the right lateral ventricle suggesting that the whole cerebral hemisphere was involved. At 3 weeks following treatment, the brains of 5 of 7 rats were microscopically positive for tumor, but only one of these animals had macroscopically visible (1–2 mm) tumor, extending from the wall of the lateral ventricle to the cortical surface. At 4 weeks, 2 of 8 rats presumptively had died of their tumors, based on a 20% loss of body weight. The remaining 6 rats all showed microscopic evidence of tumor, and in 3 animals the tumors were macroscopically evident. It is noteworthy that the microscopic tumor deposits were too small to be detected by magnetic resonance imaging (MRI), where the limit of resolution is >1 mm, which would have been equivalent to 10^6^ tumor cells. Therefore, neuropathologic examination was a more powerful approach to detect a much smaller tumor cell burden. In contrast to the rats that received both carboplatin and X-irradiation, the brains of animals that received a fractionated 20 Gy dose of X-irradiation alone all had macroscopic tumors at the time of death (3, 3, 5, and 7 mm in greatest dimension on days 31, 34, 37, and 39, respectively).

## Discussion

As previously reported by us [9], a 20 μg dose of carboplatin administered by CED over 30 min, or an 84 μg dose given over 7 d by Alzet pumps, in combination with X-irradiation, were shown to be non-neurotoxic. Doubling the dose of carboplatin to 40 μg by CED or 168 μg by Alzet pump ifusion was associated with significant neurotoxicity [9]. Therefore, the lower doses were selected for the studies described in the present study. Similarly, in a previous study using the F98 glioma model [16] we observed that an X-ray dose of 22.5 Gy, delivered to the whole brain, resulted in a 30% rate of radiation related deaths. Therefore, we chose a maximum dose of 20 Gy, delivered to the tumor bearing cerebral hemisphere, in four consecutive 5 Gy fractions. As shown in the present study, this was well tolerated. Based on the previous findings [9], in the present study the dose of carboplatin was set at either 20 μg by CED or 84 μg by Alzet pump delivery in combination with a maximum radiation dose of 20 Gy to the tumor bearing cerebral hemisphere. However, in order to determine if the survival data could be further improved over that previously reported by us [9], we also initiated X-irradiation at either 6 or 24 hrs following administration of carboplatin or divided it into two 10μg doses on days 1 and 3 with a 24 hr interval between drug administration by CED and X-irradiation with two 7.5 Gy fractions.

Turning next to the biodistribution data, the purpose of DMF treatment was to reduce interstitial pressure within the tumor [12] and thereby enhance the uptake and possibly the microdistribution of carboplatin. Although the differences in mean tumor carboplatin values for D alone and DMF-treated rats were not statistically significant, there was a suggestion that those animals that had received DMF had higher carboplatin values with a narrower range compared to those that did not. However, given the broad range, it would have required two groups of 102 animals each to attain a level of statistical significance (power = 0.8, α = 0.05) and it would have been highly unlikely in small cohorts of animals to demonstrate any significant differences in MSTs. Therefore, such studies were not carried out. This is not dissimilar from what was observed in the EORTC trial evaluating the combination of RT and temozolomide [17,18] where 573 patients were required to demonstrate a small difference (2.5 months) in overall median survival time. Since the reduction in interstitial pressure is transient [12], a single DMF treatment would not have been useful in increasing carboplatin concentration in animals that had received the drug by Alzet pump infusions. Because of the transiency of the effect, we elected to determine drug concentrations at a short time interval following termination of CED. However, it is possible that the differences in drug concentrations might have increased over longer time intervals if there were differences in the drug uptake and efflux.

As indicated earlier, the purpose of the present study was to determine if modification of the RT regimen and the dosing paradigm of carboplatin would result in improved survival data compared to those previously reported [5-9]. The very clear-cut answer to this question is that sustained delivery of carboplatin (84 μg in 168 μl) by Alzet pumps in combination with either a total dose of 20 Gy or, as previously reported [9], 15 Gy in three 5 Gy fractions, produced the greatest increases in MSTs (107.7 d *vs.* 111.8 d, respectively), with no treatment-related deaths. However, this might have been due to the higher total dose of the drug and its improved tumor microdistribution. Increasing the radiation dose to 20 Gy and administering carboplatin by CED resulted in an identical MST as that previously reported by us (83.6 d *vs.* 83.4 d, respectively) [9]. However, as shown in Table 4, the 20 Gy dose would have resulted in 35 Gy to late responding tissues such as vascular endothelium compared to 26.25 Gy with the 15 Gy dose. It is noteworthy that an additional dose of 6.25 Gy of 6 MV photons to the tumor, at conventional 2 Gy fractions (Table 4), did not have any effect on the MST, suggesting that major determinant of efficacy was drug rather than radiation-related. As previously reported [16], rats that received an X-ray dose of 22.5 Gy in three 7.5 Gy fractions had a MST of 53.2 ± 4.6 d with significant radiation-related morbidity. Modifying the carboplatin dosing paradigm to two 10 μg doses and the RT regimen to two 7.5 Gy fractions resulted in an almost identical MST (82.1 d *vs.* 83.4 d) as that previously reported [9]. Two possible ways that this combination therapy might be improved would be to increase tumor uptake and the microdistribution of carboplatin or to administer a neutralizing agent to decrease systemic toxicity associated with a higher dose of chemotherapy, as previously reported with “RADPLAT” in the treatment of advanced squamous carcinomas of the head and neck [19]. Although DMF treatment marginally increased the tumor drug concentration, it is unlikely that this would have resulted in a significant increase in MST compared to animals that did not receive DMF treatment.

The major problem, which has limited the strong synergy of RT in combination with i.c. administration of carboplatin [9], is the tremendous variability following CED of the drug (Table 1). We hypothesize that those animals that were cured of their tumors had higher tumor concentrations and better microdistributions of carboplatin. Although there may have been some differences in the sizes of the tumors at the time that the drug was administered, this does not seem to be an adequate explanation for the broad ranges in tumor carboplatin concentrations that were observed. If this is the case for a rat brain tumor model where each tumor has a volume of ~25–30 mm^3^ in a cerebral hemisphere that weighs 600 mg [9], the problem is orders of magnitude greater in the case of a patient with a highly infiltrative glioma in a cerebral hemisphere that weighs ~600 g. Although CED is superior to direct intratumoral injection [20-24], it is still a work in progress [20], and more likely than not it can be significantly improved. As has been reported previously by us [9], a 20 μg dose of carboplatin administered i.c. by CED resulted in a tumor drug concentration of 10.4 μg/g, which was equal to that attained by a dose of 20 mg (20,000 μg) administered intravenously. The major question that must be addressed, therefore, is, “How can administration of a therapeutic agent to a brain tumor be improved to achieve a higher and more homogeneous distribution of various therapeutic agents?” Basically this is a problem that will require diverse expertise to solve but it is not unreasonable to expect that such improvements will be made and that CED ultimately will prove to be highly effective clinically [25,26].

## Conclusions

Our results provide strong *proof-of-principle* that i.c. CED of carboplatin alone or in combination with X-irradiation can result in significant prolongations in MSTs and cures of a brain tumor that has been incurable by all other therapeutic strategies except for one [15]. As a first step in clinically translating these findings a Phase I trial currently is in progress at The Ohio State University to evaluate i.c. CED of carboplatin (72 ml over 3 days) in patients with recurrent high grade gliomas.

## Abbreviations

RT: Radiation therapy; DMF: Dexamethasone, mannitol, and furosemide; CED: Convection enhanced delivery; LINAC: Linear accelerator; MST: Mean survival time; EORTC: European Organization for Research and Treatment of Cancer; BBB: Blood-brain barrier; i.c.: Intracerebral; i.v.: Intravenenous; i.p.: Intraperitoneal; ICP-OES: Inductively coupled plasma – Optical emission spectroscopy; SE: Standard error; MST: Mean survival time; MeST: Median survival time; SD: Standard deviation; ANOVA: Analysis of variance; MRI: Magnetic resonance imaging.

## Competing interests

The authors declare that there are no competing interests.

## Authors’ contributions

WY: Carried out all of the animal studies and contributed to the design of experiments, and the evaluation of data. RFB: Had overall responsibility for the project, evaluated data and wrote almost the entire manuscript. TH: Assisted in carrying out animal studies, performed carboplatin determinations, analyzed the data and wrote the section on statistical analysis; RJN: Assisted in carrying out the animal studies. MW: Carried out the animal radiations with the assistance of NG, WY, TH and RJN. LA and RFB reviewed brain slides and wrote the section on neuropathologic evaluation. JCG contributed to the design of experiments and writing the manuscript. All authors read and approved the final manuscript.
